# A Self-Regulating Power-Control Scheme Using Reinforcement Learning for D2D Communication Networks

**DOI:** 10.3390/s22134894

**Published:** 2022-06-29

**Authors:** Tae-Won Ban

**Affiliations:** Department of Intelligent Communication Engineering, Gyeongsang National University, Marine Science Bldg 807, Tongyeong-si 53064, Korea; twban35@gnu.ac.kr

**Keywords:** device to device (D2D), deep deterministic policy gradient (DDPG), deep reinforcement learning (DRL), power control

## Abstract

We investigate a power control problem for overlay device-to-device (D2D) communication networks relying on a deep deterministic policy gradient (DDPG), which is a model-free off-policy algorithm for learning continuous actions such as transmitting power levels. We propose a DDPG-based self-regulating power control scheme whereby each D2D transmitter can autonomously determine its transmission power level with only local channel gains that can be measured from the sounding symbols transmitted by D2D receivers. The performance of the proposed scheme is analyzed in terms of average sum-rate and energy efficiency and compared to several conventional schemes. Our numerical results show that the proposed scheme increases the average sum-rate compared to the conventional schemes, even with severe interference caused by increasing the number of D2D pairs or high transmission power, and the proposed scheme has the highest energy efficiency.

## 1. Introduction

Device-to-device (D2D) communication has become an attractive solution as one of many promising technologies for next-generation mobile communication networks, as it can significantly increase spectral efficiency and also enables direct communication of mobile devices when the mobile communication signal is unavailable or base stations (BSs) are not accessible in disaster situations [1,2]. In addition, it can provide various direct connectivities for sensor devices without cellular infrastructure [3]. In D2D communication networks, the simultaneous transmission of multiple transmitters can cause serious interference, which is one of the challenging problems that hinder the prevalence of D2D communication networks. Therefore, there is inevitably a need to reduce inter-link interference by power control. Many power-control algorithms have been proposed that rely on conventional mathematical approaches [4,5,6,7,8,9,10]. Despite intensive investigations on the power control problem in D2D communication networks, the closed-form solutions of general power control problems to maximize the sum-rate of D2D communication networks in which multiple D2D links share the same radio resource are not available, as they are typically NP-hard. As an alternative, new power-control schemes have prepared to overcome the limitations of conventional schemes using deep learning have been proposed [11,12,13,14,15,16]. However, they unfortunately do not allow each D2D user to autonomously determine its transmission power level because cellular base stations (BSs) play a key role in coordinating the transmission power levels of cellular and D2D users or each D2D pair needs to collect not only local information that can be obtained directly by the transmitter or receiver of a D2D pair but also non-local information that can be obtained from neighboring D2D pairs, thereby causing extra signaling overhead.

In this paper, we also investigate an overlay D2D communication network and propose a fully distributed power control algorithm based on deep learning, with which each D2D transmitter can determine its transmission power by using local interference information directly obtained by measuring sounding signals from D2D receivers. The proposed scheme uses a deep deterministic policy gradient (DDPG) that supports continuous action spaces such as transmission power levels. The performance of the proposed scheme is analyzed in terms of average sum-rates and energy efficiency and is compared to that of reference schemes including FPLinQ. FPLinQ can be a good comparison target as in other studies because it is difficult to reproduce DRL-based simulations in previous studies due to the lack of detailed information on simulation environments such as the structure of deep learning networks and many hyper-parameters. Furthermore, FPLinQ has been shown to outperform many DRL-based power control schemes through its iterative optimization. Our numerical results show that the average sum-rate of the proposed scheme is always comparable or superior to the highest one obtained by the best-performing reference scheme. In addition, the average sum-rate of the proposed scheme improves as the number of D2D pairs increases, while the average sum-rate of all reference schemes deteriorates. It is also shown that the proposed scheme has the highest energy efficiency compared to all reference schemes. More specifically, the proposed scheme can achieve 168∼506% of average energy efficiency obtained by the best performing reference scheme when the number of D2D pairs is 50. The rest of this paper is organized as follows. We investigate related works in Section 2. In Section 3, a D2D communication network and channel model examined in this paper are described. A distributed power control scheme using DDPG is proposed in Section 4. Section 5 presents the numerical results used to analyze the performance of the proposed scheme. Finally, the conclusions of this paper are drawn in Section 6.

## 2. Related Works

Many power control algorithms based on conventional numerical or heuristic approaches have been proposed to resolve the interference problem in D2D networks [4,5,6,7,8,9,10]. A power control scheme for full-duplex D2D communications underlying cellular networks was proposed based on a high signal-to-interference-noise ratio (SINR) approximation [4]. Another power control scheme was also proposed for cellular multiple antenna networks based on an iterative approach [5], which has been widely applied to D2D communication networks due to the similarity between the two networks. Binary power control schemes were proposed to reduce the computational complexity, preserving the performance [6,7]. In the FlashLinQ, each D2D communication link is activated for data transmission only when the link generates interference lower than a predetermined threshold to keep the total amount of interference below a certain level, and the threshold should be optimized for a given environment, which is the critical drawback of FlashLinQ [6]. The binary link activation problem was reformulated into a fractional programming form in [7] and a new optimization strategy called fractional-programming-based link scheduling (FPLinQ) was created. Compared to FlashLinQ, FPLinQ does not require the optimization of threshold values and thereby shows a significant performance improvement. However, FPLinQ requires a central node to collect all channel gains and to coordinate link-activation decisions in an iterative approach, which necessarily causes a heavy signaling overhead and computational complexity. A power control problem for D2D communication networks using a two-way amplify-and-forward (AF) relay was investigated in [8], where the power control problem was formulated as an optimization problem and solved using an iterative approach. A joint problem of resource allocation and power control for cellular assisted D2D networks was investigated, and an efficient framework was proposed to maximize the number of supported links [9]. D2D transmission power control schemes were proposed to maximize the D2D rate while maintaining the performance of cellular networks, and an asymptotic upper bound on the D2D rates of the proposed schemes was provided [10].

On the other hand, new power-control schemes based on deep learning for D2D networks have been proposed to overcome the limitations of the conventional schemes such as optimization of threshold values, computational complexity, or signaling overhead [11,12,13,14,15,16]. Deep reinforcement learning (DRL)-based power control schemes for D2D communications underlying cellular networks were investigated [11,12,13]. A joint scheme for resource block scheduling and power control to improve the sum-rate of D2D underlay communication networks was proposed based on a deep Q-network considering users’ fairness [11]. However, this proposed scheme requires coordination by cellular base stations. A deep-learning-based transmission power allocation method was proposed to automatically determine the optimal transmission powers for D2D networks underlying full duplex cellular networks [12]. It was shown that the performance of the proposed scheme is comparable with that of the traditional iterative algorithms, but the intervention of cellular base stations is also required. A centralized DRL algorithm to solve the power allocation problem of D2D communications in time-varying environments was proposed in [13]. The proposed algorithm considers a D2D network as a multi-agent system and represents a wireless channel as a Finite-State Markov Channel (FSMC).

Although underlay D2D communications can significantly enhance overall spectral efficiency, the quality of cellular communications cannot be tightly guaranteed because of the cross-interference caused by D2D communications. Thus, deep-learning-based power control schemes for overlay D2D communication systems were proposed in [14,15,16]. Cellular and D2D users utilize different radio resources that are orthogonal to each other in order to guarantee the quality of cellular communications by avoiding the cross-interference. A joint channel selection and power -control optimization problem was investigated with the aim of maximizing the weighted sum-rate of D2D networks and a distributed deep-reinforcement-learning-based scheme exploiting local information and outdated nonlocal information was proposed [14]. However, this proposed scheme does not outperform the conventional algorithm based on fractional programming, and it requires global channel state information, although it is outdated [14]. A deep-learning-based power control scheme using partial and outdated channel state information was proposed in [15]. This proposed scheme achieved better spectral efficiency and energy efficiency with reduced complexity and latency compared to the iterative conventional power allocation scheme. However, cellular BSs are also required to collect channel state information for D2D links, compute transmission power allocation levels, and inform the power allocation information of D2D transmitters. Another distributed deep learning method for power control in overlay D2D networks was proposed in [16]. This scheme predicts the real-time interference pattern from the outdated interference information and makes a decision for power allocation by using a recurrent neural network (RNN). This scheme also requires each D2D pair to collect non-local information from all the D2D pairs to determine its transmission power, as in the scheme proposed in [14]. Even though the performance was analyzed in highly correlated channel environments where the prediction of interference pattern is relatively accurate, the performance was still lower than that of FPLinQ using real-time information.

## 3. A D2D Communication Network and Channel Model

**Figure 1:**
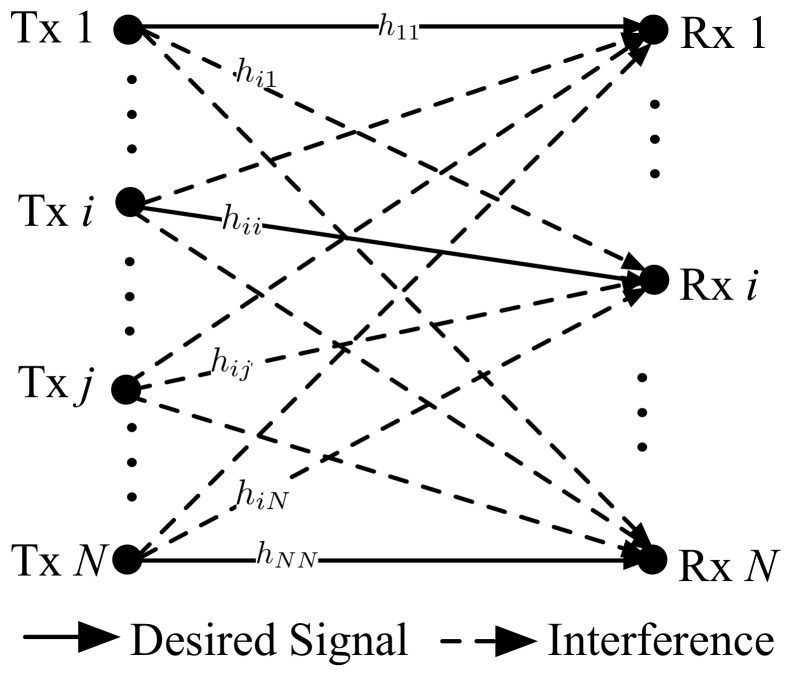
An example of an overlay D2D communication network with *N* D2D pairs.

Figure 1 illustrates an overlay D2D communication network in which D2D communications use extra radio resources orthogonal to those used by cellular communications. We have *N* D2D pairs, and each D2D transmitter transmits data to its corresponding receiver by sharing the same radio resource. Let $h_{ij}$ denote the channel coefficient between transmitter *j* and receiver *i*. If i=j, $h_{ij}$ denotes the coefficient of the desired signal that transmitter *i* transmits to its paired receiver *i*. Otherwise, $h_{ij}$ denotes the coefficient of the interfering signal that transmitter *j* generates to the receiver *i*. We consider a semi-static block fading channel model in which all channel coefficients are static during the data transmission intervals and randomly vary during every data transmission interval. Rayleigh channel fading is considered, and all channel coefficients follow a complex Gaussian distribution ∼CN(0,1). In addition, we assume that all channel coefficients are independent and identically distributed. D2D communications use time-division duplex (TDD) as a duplex scheme. It is also assumed that $h_{ij}=h_{ji}\;\forall i,\;j$ because of the channel reciprocity of TDD without loss of generality. All D2D transmitters have a peak transmission power constraint *P*, and $p_{i}\left(0\leq p_{i}\leq P\;\forall i\right)$ denotes an instantaneous transmission power level of D2D transmitter *i*. The signal-to-interference-and-noise ratio (SINR) perceived at the D2D receiver *i* can be calculated as $\left(1+\frac{p_{i}{\left|h_{ii}\right|}^{2}}{\sum_{j=1,j\neq i}^{N}p_{j}{\left|h_{ij}\right|}^{2}+N_{0}}\right)$. Then, the sum-rate of the D2D network shown in Figure 1 can be given by

(1) $$\begin{array}{cc} r\; & =\sum_{i=1}^{N}\log_{2}\left(1+\frac{p_{i}{\left|h_{ii}\right|}^{2}}{\sum_{j=1,j\neq i}^{N}p_{j}{\left|h_{ij}\right|}^{2}+N_{0}}\right), \end{array}$$

where $N_{0}$ denotes the thermal noise power. Our goal is to achieve self-regulation of the transmission power $p_{i}$ in a distributed manner for each D2D transmitter *i* in order to maximize the sum-rate *r*.

## 4. Proposed Power Control Scheme

**Figure 2:**
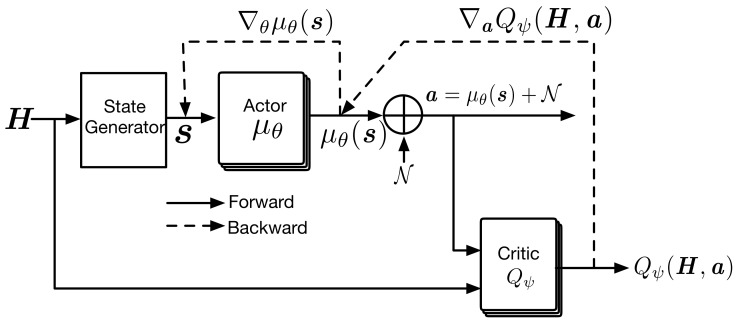
Architecture for training DDPG in the proposed power control scheme.

Figure 2 shows the architecture for training the DDPG-based DRL network in the proposed power control scheme, which consists of the Actor network μ with parameters θ and Critic network *Q* with parameters ψ. H is the matrix of channel gains. The (i,j) entry of H is $|h_{ij}{|}^{2}$ and $H\in R^{N\times N}$. The state generator builds N×N matrix s, described by

(2) $$s=\left[\begin{array}{c} s_{1}\\ \vdots \\ s_{i}\\ \vdots \\ s_{N} \end{array}\right]=\left[\begin{array}{cccc} |h_{11}{|}^{2} & |h_{21}{|}^{2} & \cdots & |h_{N1}{|}^{2}\\ \; & \; & \vdots & \\ |h_{ii}{|}^{2} & |h_{2i}{|}^{2} & \cdots & |h_{Ni}{|}^{2}\\ \; & \; & \vdots & \\ |h_{NN}{|}^{2} & |h_{2N}{|}^{2} & \cdots & |h_{(N-1)N}{|}^{2} \end{array}\right].$$

s consists of *N* row vectors, $s_{1},\cdots ,s_{i},\cdots s_{N}$. The input state for the D2D transmitter *i* denoted by $s_{i}$ consists of the gain of the desired link and (N−1) gains of interference links that the transmitter *i* generates toward other receivers and is given by

(3) $$\begin{array}{c} s_{i}=\left[|h_{ii}{|}^{2}\;|h_{2i}{|}^{2}\cdots \;{\left|h_{Ni}\right|}^{2}\right]. \end{array}$$

Contrary to the conventional DRL-based power control schemes, the proposed scheme composes the $s_{i}$ only of the local channel gains that each transmitter can obtain by measuring sounding symbols transmitted by receivers without extra feedback from other transmitters. In addition, the gain in the desired link is set in the first place regardless of *i*, followed by the gains in interference links to preserve the context of $s_{i}\;\forall i$ and to enable distributed operation after the completion of training. In order to train the DDPG network, the Actor $\mu_{\theta }$ takes the input matrix s as the input and yields the output $\mu_{\theta }\left(s\right)$, which is a column vector with *N* elements and can be interpreted as actions of *N* transmitters. The Actor consists of three fully connected layers with 128, 64, and 1 neuron(s), respectively. The first two layers are activated by rectified linear unit (ReLU), and the last layer is activated by $\frac{(\tanh(\cdot )+1)P}{2}$ so that the final output $\mu_{\theta }\left(s\right)$ satisfies $0\leq \mu_{\theta }\left(s\right)\leq P$. The random noise is added to $\mu_{\theta }\left(s\right)$ to make the DDPG policies explore better during training. We use an Ornstein–Uhlenbeck process to generate the random noise, as in the original DDPG paper [17], where random noise N is sampled from a correlated normal distribution. The final actions of *N* transmitters are determined by $a={\left[p_{1}\cdots p_{N}\right]}^{T}=\mu_{\theta }\left(s\right)+N$, which are the transmission power levels of *N* transmitters.

For training Critic $Q_{\psi }$, actions a and channel matrix H are forwarded to Critic $Q_{\psi }$, which consists of two fully connected layers of size 64 and 1 activated by ReLU, and the final output $Q_{\psi }\left(H,a\right)$ is calculated. The $s_{i}$ consists only of the local channel gains to allow a fully distributed operation according to the proposed scheme. The s is not sufficient to exactly evaluate the value of rewards generated by transmitters’ actions. Thus, H is used as the input of the Critic instead of s in order to evaluate exactly the transmitters’ actions. However, it is notable that the Critic is only necessary during the training process. H is unnecessary, and $s_{i}$ is sufficient for transmitter *i* to determine its transmission power with the trained Actor network in the execution process. The target value of the Critic network can be calculated by

(4) $$\begin{array}{c} \hat{Q}=r+\lambda Q_{\psi^{t}}^{t}\left(H^{\prime },\mu_{\theta^{t}}^{t}\left(s^{\prime }\right)\right), \end{array}$$

where *r*, λ, $Q_{\psi^{t}}^{t}$, $\mu_{\theta^{t}}^{t}$, and $s^{\prime }$ denote the sum-rate for given H and a, a discounting factor for future rewards, target Critic network, target Actor network, and new state caused by a, respectively. In this paper, s and a consist of channel gains and transmission power levels, respectively, and $s^{\prime }$ is independent of a. Thus, λ can be set to 0, and target networks are unnecessary for our considerations. The update of parameters takes place in two stages. The loss of the Critic network is defined by

(5) $$\begin{array}{c} L_{Q}=E_{H}\left[{\left(\hat{Q}-Q_{\psi }\left(H,a\right)\right)}^{2}\right]. \end{array}$$

The parameters of the Critic can be easily updated to minimize the loss $L_{Q}$ because the Actor network can be considered constant. Then, it is straightforward to calculate the gradient of $L_{Q}$ with respect to ψ. The loss of the Actor network is defined by

(6) $$\begin{array}{c} L_{\mu }=-E_{H}\left[Q_{\psi }\left(H,\mu_{\theta }\left(s\right)\right)\right]. \end{array}$$

We need to train the deterministic policy $\mu_{\theta }\left(s\right)$ to generate actions that maximize $Q_{\psi }\left(H,\mu_{\theta }\left(s\right)\right)$, where $\mu_{\theta }\left(s\right)$ is contained inside $Q_{\psi }$. Thus, the gradient of $L_{\mu }$ with respect to θ can be calculated as

(7) $$\begin{array}{c} \nabla_{\theta }L_{\mu }=E_{H}\left[\nabla_{\theta }\mu_{\theta }\left(s\right)\times \nabla_{a}Q_{\psi }\left(H,a\right)|a=\mu_{\theta }\left(s\right)\right] \end{array}$$

using the chain rule. The parameters of the Actor network are updated by a gradient descent by treating the parameters of the Critic network as constants. When the parameters’ training is completed, each D2D transmitter is only equipped with the Actor without a Critic and will be provided with the trained parameters for the Actor network. In addition, the Actor’s parameters can be periodically updated by over-the-air (OTA) or a firmware update. Moreover, each D2D transmitter can easily build its input states by measuring sounding symbols from surrounding D2D receivers. The overall procedures of the proposed power control scheme using DDPG is summarized in Algorithm 1. In addition, after the training is complete, each D2D transmitter only executes the lines 4∼6, 8, and 9, which are in italics.
**Algorithm 1** Proposed power control algorithm using DDPG1:Initialize all parameters2:Generate Actor and Critic networks3:**while** episode < MAX_EPISODES **do**4:       *Generate channel gains H for the D2D network shown in Figure 1*5:       *Build the input state s using (2)*6:       *Calculate $\mu_{\theta }\left(s\right)$ using Actor network*7:       Generate random noise N for exploration8:       *Determine the final action*9:       *D2D transmitters transmit data with the power levels set by the determined final actions*10:     Calculate the reward using (1)11:     Calculate $Q_{\psi }\left(H,a\right)$ using Critic network12:     Calculate the losses of Critic and Actor networks using (5) and (6)13:     Calculate the gradients of $\nabla_{\psi }L_{Q}$ and $\nabla_{\theta }L_{\mu }$14:     Update the parameters of Critic and Actor networks using $\nabla_{\psi }L_{Q}$ and $\nabla_{\theta }L_{\mu }$15:     episode += 116:**end while**

## 5. Numerical Results

**Figure 3:**
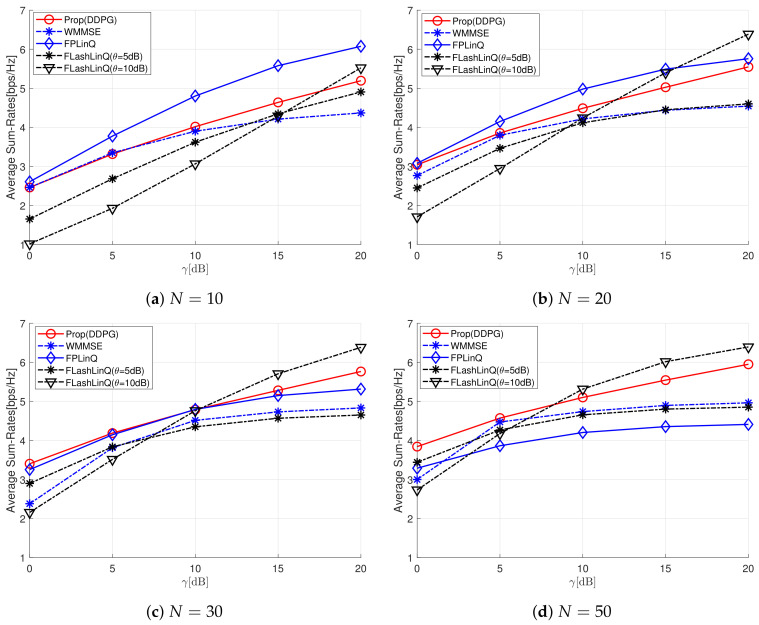
Average sum-rate for various γ and *N* values.

**Figure 4:**
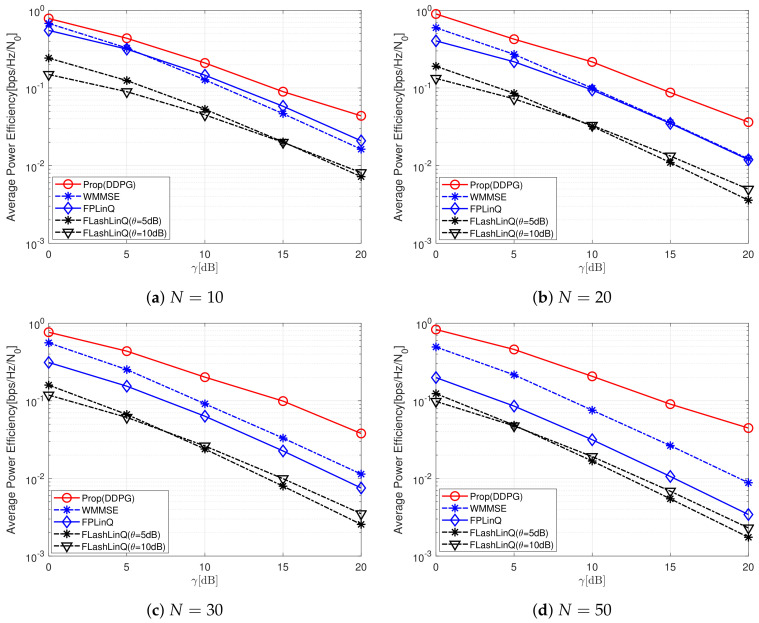
Average energy efficiency for various γ and *N* values.

We investigate the performance of the proposed power control scheme using DDPG and compare it with the reference schemes in Figure 3 and Figure 4 and Table 1 and Table 2. The reference schemes include weighted minimum mean square error (WMMSE), FPLinQ, and FLashLinQ. WMMSE is typically used to tackle NP-hard power control problems in an iterative manner due to its superiority [5]. The performance of all the schemes is analyzed in terms of average sum-rate and energy efficiency for varying maximal peak transmission power and the number of D2D pairs. For a mathematical simplification, the maximal peak transmission power *P* is normalized with respect to the thermal noise power $N_{0}$, and the normalized maximal peak transmission power is defined by $\gamma =\frac{P}{N_{0}}$.

Figure 3a shows the average sum-rates for varying γ when N=10. For a given γ, FLashLinQ shows the different average sum-rates according to θ, which is a threshold determining whether to transmit data. For γ>15 dB, a high θ yields a high average sum-rate, and a lower θ yields a high average sum-rate for γ<15 dB. The average sum-rate of WMMSE is higher than that of FLashLinQ for γ<15 dB, and vice versa for γ≥15 dB. The proposed scheme outperforms WMMSE and FLashLinQ except for γ=20 dB. Even though FlashLinQ with θ=10 dB outperforms the proposed scheme for γ=20 dB, its average sum rates is the lowest for γ<15 dB among all the schemes. FPLinQ outperforms all the other schemes regardless of γ, which shows that FPLinQ works well when *N* is small. Figure 3b shows the average sum-rate for N=20. Compared to N=10, the average sum-rates of WMMSE, FPLinQ, and FLashLinQ with θ=5 dB all increase if γ≤10 dB and decrease for γ>10 dB because they cannot cope well with the severe cross-interference caused by increasing *N* and γ. However, the average sum-rates of the proposed scheme and FLashLinQ with θ=10 dB continuously increase even if γ>10 dB, thereby showing that the both schemes are capable of coping well with severe cross-interference. Figure 3c shows that the proposed scheme begins to outperform FPLinQ when N=30 and is superior to all the reference schemes except for FLashLinQ with θ=10. FLashLinQ with θ=10 dB has the highest average sum-rate if γ>10 dB because it is optimal for a single D2D pair with the highest channel gain to transmit data in interference-limited environments [18]. FLashLinQ with a higher threshold θ reduces the number of D2D pairs to transmit data simultaneously. However, its average sum-rate is seriously degraded if γ<10 dB because it is optimal for all D2D pairs to transmit data in power-limited environments, but D2D pairs are suppressed from transmitting data because of the high threshold. Figure 3d shows that the tendency shown in Figure 3c becomes more pronounced as *N* increases up to 50. The average sum-rate of FPLinQ is seriously degraded, while the average sum-rate of the proposed scheme is greatly enhanced. Table 1 shows the average sum-rate ratio of the proposed scheme to the best performing reference scheme. The schemes in parentheses denote the reference scheme with the highest average sum-rate for a given γ and *N*. The best-performing reference scheme varies according to γ and *N* values, and the average sum-rates of the proposed scheme improves as *N* increases. If 0≤γ≤5 and N=50, the proposed scheme outperforms the best-performing reference scheme by 2∼12%. Otherwise, the average sum-rate of the proposed scheme is comparable to the highest average sum-rate obtained by the best-performing reference scheme. It is also shown that the difference in average sum-rate between the proposed scheme and the best-performing reference scheme decreases as *N* increases or γ decreases. When N=50, the proposed scheme can achieve 112% and 93% of the average sum-rate obtained by the best-performing reference scheme for γ=0 dB and γ=20 dB, respectively.

On the other hand, energy efficiency is also one of import performance metrics for communication networks, and instantaneous transmission power levels of all D2D transmitters vary according to power control schemes. Accordingly, we also investigate the energy efficiency of all schemes. We normalize the average sum-rate with respect to the average power consumption to calculate the average sum-rate that can be obtained with a transmission power level equal to $N_{0}$. The results of energy efficiency are presented in Figure 4a–d. Although FLashLinQ outperforms the proposed scheme in terms of average sum-rate in interference-limited environments with high values of *N* and γ, its energy efficiency is the lowest among all schemes. The energy efficiency of FPLinQ is similar to that of WMMSE when *N* = 10 or 20, and it is also seriously degraded as *N* increases above 20 and becomes lower than that of WMMSE. As *N* increases, the energy efficiency of the proposed scheme improves regardless of γ, while the energy efficiency of all the reference schemes deteriorates. Table 2 shows the average energy-efficiency ratio of the proposed scheme to the highest one obtained by the reference schemes. The schemes in parentheses also denote the reference scheme with the highest energy efficiency. If 10≤γ≤20 and 10≤N≤20, FPLinQ has the highest energy efficiency among the reference schemes. Otherwise, WWMSE has the highest energy efficiency among the reference schemes. The proposed scheme has the highest energy efficiency compared to all reference schemes. For N=50, the proposed scheme can achieve 168∼ 506% of average energy efficiency obtained by the best-performing reference scheme.

## 6. Conclusions

In this paper, we propose a self-regulating power control scheme based on deep reinforcement learning for D2D communication networks. The proposed scheme uses DDPG to generate a continuous action, which corresponds to the transmission power level of each D2D transmitter. The DDPG uses full channel gains as an input state to the Critic network in order to evaluate the actions performed by each D2D transmitter during the training phase, but it only uses local channel gains that each D2D transmitter can obtain by measuring the uplink sounding symbols transmitted by surrounding D2D receivers as an input state to the Actor network. Thus, each D2D transmitter can autonomously determine its transmission power level upon training completion. The performance of the proposed power control scheme is compared to the other reference schemes such as FLashLinQ, FPLinQ, and WMMSE in terms of average sum-rate and energy efficiency. The average sum-rate in the proposed scheme begins to be higher than in the reference schemes when *N* increases beyond 20. Moreover, the presented scheme has the highest energy efficiency in all situations. It can be concluded that the proposed scheme allows D2D pairs to deal with severe interference in large-scaled D2D networks with a large number of D2D pairs by self-regulating their transmission power levels while achieving high energy efficiency.

## Figures and Tables

**Table 1:** The average sum-rate ratio of the proposed scheme to the best-performing reference scheme. The schemes in parentheses denote the reference scheme with the highest average sum-rate.

γ [dB]	*N*			
	10	20	30	50
0	0.94 (FPLinQ)	0.99 (FPLinQ)	1.05 (FPLinQ)	1.12 (FPLinQ)
5	0.88 (FPLinQ)	0.93 (FPLinQ)	1.01 (FPLinQ)	1.02 (WMMSE)
10	0.84 (FPLinQ)	0.90 (FPLinQ)	1.00 (FPLinQ)	0.96 (FLashLinQ)
15	0.83 (FPLinQ)	0.92 (FPLinQ)	0.93 (FPLinQ)	0.92 (FLashLinQ)
20	0.85 (FPLinQ)	0.87 (FPLinQ)	0.90 (FLashLinQ)	0.93 (FLashLinQ)

**Table 2:** The average energy-efficiency ratio of the proposed scheme to the best performing reference scheme. The schemes in parentheses also denote the reference scheme with the highest energy efficiency.

γ [dB]	*N*			
	10	20	30	50
0	1.16 (WMMSE)	1.51 (WMMSE)	1.37 (WMMSE)	1.68 (WMMSE)
5	1.33 (WMMSE)	1.57 (WMMSE)	1.73 (WMMSE)	2.12 (WMMSE)
10	1.44 (FPLinQ)	2.18 (FPLinQ)	2.21 (WMMSE)	2.74 (WMMSE)
15	1.54 (FPLinQ)	2.44 (FPLinQ)	2.99 (WMMSE)	3.43 (WMMSE)
20	2.09 (FPLinQ)	2.98 (FPLinQ)	3.32 (WMMSE)	5.06 (WMMSE)

## Data Availability

Not applicable.
